# Leveraging machine learning models in evaluating ADMET properties for drug discovery and development

**DOI:** 10.5599/admet.2772

**Published:** 2025-06-07

**Authors:** Magesh Venkataraman, Gopi Chand Rao, Jeevan Karthik Madavareddi, Srinivas Rao Maddi

**Affiliations:** Department of Pharmacology, Acubiosys Private Limited, Hyderabad, Telangana, India

**Keywords:** ADMET prediction, AI/ML, pharmacokinetics, computational toxicology, molecular descriptors

## Abstract

**Background and purpose:**

The evaluation of ADMET properties remains a critical bottleneck in drug discovery and development, contributing significantly to the high attrition rate of drug candidates. Traditional experimental approaches are often time-consuming, cost-intensive, and limited in scalability. This review aims to investigate how recent advances in machine learning (ML) models are revolutionizing ADMET prediction by enhancing accuracy, reducing experimental burden, and accelerating decision-making during early-stage drug development.

**Experimental approach:**

This article systematically examines the current landscape of ML applications in ADMET prediction, including the types of algorithms employed, common molecular descriptors and datasets used, and model development workflows. It also explores public databases, model evaluation metrics, and regulatory considerations relevant to computational toxicology. Emphasis is placed on supervised and deep learning techniques, model validation strategies, and the challenges of data imbalance and model interpretability.

**Key results:**

ML-based models have demonstrated significant promise in predicting key ADMET endpoints, outperforming some traditional quantitative structure - activity relationship (QSAR) models. These approaches provide rapid, cost-effective, and reproducible alternatives that integrate seamlessly with existing drug discovery pipelines. Case studies discussed in this review illustrate the successful deployment of ML models for solubility, permeability, metabolism, and toxicity predictions.

**Conclusion:**

Machine learning has emerged as a transformative tool in ADMET prediction, offering new opportunities for early risk assessment and compound prioritization. While challenges such as data quality, algorithm transparency, and regulatory acceptance persist, continued integration of ML with experimental pharmacology holds the potential to substantially improve drug development efficiency and reduce late-stage failures.

## Introduction

The typical timeframe for drug discovery and development of a new drug spans from 10 to 15 years of rigorous research and testing [1]. The sheer volume of potential drug candidates renders traditional wet lab experiments impractical. However, advancements in data science over the past decade, coupled with enhanced computational capabilities, have paved the way for in silico methodologies for screening extensive drug libraries. This preliminary step, preceding preclinical studies, significantly reduces costs and expands the scope of drug discovery efforts [2]. Machine learning (ML) is a method of data analysis involving the of new algorithms and models capable of interpreting a multitude of data. Within this framework, ML techniques have emerged as pivotal tools in the pharmaceutical drug discovery and development field [3]. Recent progress in ML algorithms, coupled with the accessibility of extensive proprietary and public absorption, distribution, metabolism, excretion and toxicity (ADMET) datasets, has sparked enthusiasm among academic and pharmaceutical science circles in predicting pharmacokinetic and physicochemical endpoints in early drug discovery [4].

**Figure 1.**
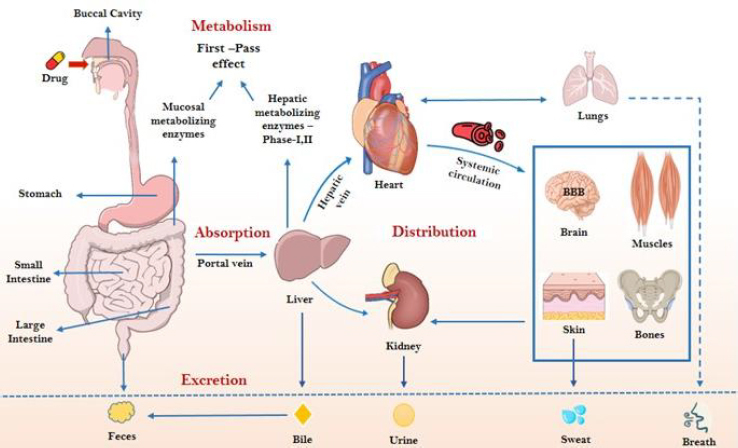
This figure depicts the absorption, distribution, metabolism, and excretion (ADME) process for drugs administered orally. When a tablet or capsule is ingested, it disintegrates in the gastrointestinal (GI) tract, releasing drug molecules. These molecules either dissolve for absorption or remain in a precipitated state, eventually being excreted. Absorbed drug molecules must cross the gut wall, where they may be transported back into the intestinal lumen or metabolized by enzymes. Those that successfully traverse the gut barrier enter the liver via the portal circulation. The liver plays a crucial role in drug metabolism, utilizing phase I (modification) and phase II (conjugation) enzymatic reactions to increase the hydrophilicity of xenobiotics, facilitating their elimination through the kidneys. Drugs that escape metabolism enter systemic circulation, though a portion binds to plasma proteins, limiting their bioavailability. Only the free, unbound drug and its metabolites can reach target cells and interact with biomolecules to exert therapeutic effects. Meanwhile, some drug molecules are rapidly cleared by the kidneys. The drug’s efficacy is determined by its ability to reach and maintain an optimal concentration at the site of action while navigating these physiological processes *(Courtesy:NIH Bioart; Bioicons).*

It has been widely recognized that ADMET should be evaluated as early as possible [5]. Figure 1 illustrates the schematic representation of the ADME process for orally administered drugs, illustrating key pharmacokinetic phases including absorption in the gastrointestinal tract, metabolism in the liver, distribution via systemic circulation, and excretion through renal clearance. In silico ADME-Toxicity (ADME-Tox) evaluation models have been developed as an additional tool to assist medicinal chemists in the design and optimization of leads [6]. The majority of the problems arising during the process of drug discovery include unfavourable ADMET properties, which have been known to be a major cause of failure of the potential molecules in the drug development pipeline, contributing to large consumption of time, capital and human resources [7].

This has increased the interest in the early-stage prediction of ADMET properties of drug candidates so that the success rate of a compound reaching the later stages of drug development can be enhanced. ML has been effectively utilized to develop models and prediction tools for ADMET properties. Apart from property predictions, ML has also contributed to early phases of drug discovery, like de novo designing of chemical compounds and peptides [8]. Moreover, companies involved in clinical research have ascertained that revising the research strategies by introducing ML-based techniques has resulted in greater success rates in both preclinical and clinical trials [9]. After high-throughput screening, the chosen compounds, often referred to as hit compounds, are analysed for their biological activity via in vitro studies. The most potent compounds obtained from in vitro activity data are developed into lead compounds through a lead optimization process [10]. During the lead optimization phase, the compounds are modified to improve their bioavailability, solubility, partition coefficient, and stability as these factors can have a direct impact on the drug's therapeutic efficacy and potency [11]. The molecules with optimized ADMET properties are then further evaluated for their effectiveness using suitable animal models [12].

The optimized compounds are tested in human subjects in order to validate and confirm the potency, therapeutic efficacy, ADMET and possible adverse drug reactions through a four-step process called clinical trials, in which each step is carried out in a varying number of human subjects in a randomized control manner [13].

This review mainly focuses on the ML-based tools used in ADMET properties. We have given a general introduction to the drug discovery process, Machine learning and DL techniques, followed by specific examples and discussion on various ML and artificial intelligence (AI) based tools for drug development. We also pointed out some notable success stories in the use of AI and ML in ADMET.

## 2. Fundamentals of machine learning in drug discovery

### 2.1. Basics of machine learning models

**Figure 2.**
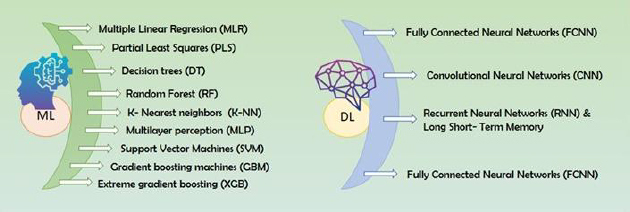
Commonly used AI/ML algorithms for developing ADMET prediction models

**Figure 3.**
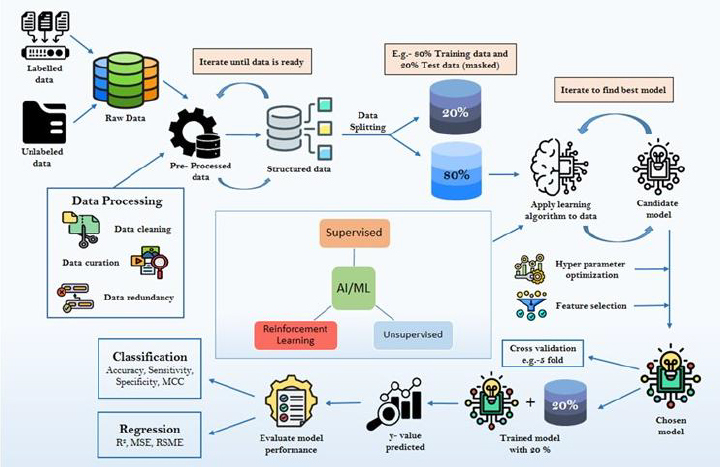
The diagram illustrates the stepwise workflow for generating a machine learning model, starting from raw data collection to model evaluation. Initially, raw data, which can include both labeled and unlabeled datasets, undergoes preprocessing and structuring. This iterative step ensures data quality before proceeding further. Once the data is refined, it is split into training and test sets, commonly in an 80:20 ratio of the total volume of the data. The 80 % training data is then subjected to various machine learning algorithms, including supervised, unsupervised, reinforcement learning, and deep learning approaches. Through an iterative process, the best-performing candidate model is selected. The chosen candidate model is further refined through hyperparameter optimization and feature selection, leading to the final optimized model. This model is then validated using cross-validation techniques such as 5-fold cross-validation to ensure robustness. Following validation, the model is tested using the 20 % test dataset. The predicted outcomes (y-values) are analysed, and the model's performance is evaluated using classification and regression metrics, ensuring its reliability and accuracy for real-world applications (Courtesy: Bioicons; Flaticon).

ML starts with obtaining a suitable dataset, often from publicly available sources, and has become increasingly prominent in ADMET prediction, offering valuable tools for drug development and toxicity [14]. ML methods are generally divided into supervised and unsupervised approaches. In supervised learning, models are trained using labelled data to make predictions, such as predicting properties like pharmacokinetic (PK) properties, based on input attributes like chemical descriptors of new compounds. On the other hand, unsupervised learning aims to find patterns, structures, or relationships within a dataset without using labelled or predefined outputs. Its goal is to uncover inherent structures and insights, which are sometimes missed in supervised learning approaches because they rely on pre-defined answers [3] Common ML algorithms used in this field include supervised methods such as support vector machines, random forests, decision trees, and neural networks, as well as unsupervised approaches like Kohonen's self-organizing maps [15] (Figure 2). These methods can be applied to various types of input data, ranging from chemical structural descriptors to transcriptome analysis, enhancing prediction accuracy [16]. The selection of appropriate ML techniques depends on the characteristics of available data and the specific ADMET property being predicted [15]. The development of a robust machine learning model for ADMET predictions begins with raw data collection, which includes both labelled and unlabelled datasets. This data undergoes preprocessing, ensuring quality and consistency before being split into training and testing datasets. Various ML algorithms, such as supervised, unsupervised, and deep learning approaches, are then applied to the training data to develop predictive models. To enhance model accuracy and generalizability, feature selection and hyperparameter optimization are performed, followed by cross-validation techniques like k-fold validation. Finally, the optimized model is tested using an independent dataset to evaluate its performance based on classification and regression metrics. Figure 3 illustrates the stepwise workflow for generating an ML model, detailing each phase from data preprocessing to model evaluation.

### 2.2. Data requirements and preprocessing

The standard ML methodology starts with obtaining a suitable dataset, often from publicly available repositories tailored for drug discovery. The quality of data is crucial for successful ML tasks, as it directly impacts model performance. Various databases provide pharmacokinetic and physicochemical properties, enabling robust model training and validation [4]. A comprehensive list of such databases is presented in Table 1. Data preprocessing, including cleaning, normalization, and feature selection, is essential for improving data quality and reducing irrelevant or redundant information [17]. Feature quality, such as relevant, informative, and predictive a specific feature is within a dataset has been shown to be more important than feature quantity, with models trained on non-redundant data achieving higher accuracy (>80 %) compared to those trained on all features [18]. When dealing with imbalanced datasets, combining feature selection and data sampling techniques can significantly improve software defect prediction performance. Empirical results suggest that feature selection based on sampled data outperforms feature selection based on original data [19]. These findings highlight the importance of carefully considering data quality, feature selection, and handling of imbalanced datasets in ML tasks to achieve optimal model performance.

**Table 1.** A list of databases that contain pharmacokinetic data for machine learning analyses

Database	Source	Ref.
PK-DB- pharmacokinetics database	https://pk-db.com/	[20]
e-Drug3D	https://chemoinfo.ipmc.cnrs.fr/MOLDB/index.php	[21]
Drugbank	https://go.drugbank.com	[22]
ChEMBL	https://www.ebi.ac.uk/chembl/	[23]
Therapeutic target database	https://db.idrblab.net/ttd/	[24]
CvTdb	https://github.com/USEPA/CompTox-PK-CvTdb	[25]
PubChem	https://pubchem.ncbi.nlm.nih.gov/	[26]
Zinc	https://zinc15.docking.org/	[27]
SuperCYP	https://insilico-cyp.charite.de/SuperCYPsPred/	[28]
The ADMET prediction database	http://modem.ucsd.edu/adme/databases/databases.htm	[29]

### 2.3. Feature engineering in ADME-tox prediction

Feature engineering plays a crucial role in improving ADMET prediction accuracy. Traditional approaches rely on fixed fingerprint, *i.e.* an efficient and quick means of portraying fixed-length data, ignoring the internal substructures within representations of molecules [30]. However, recent advancements involve learning task-specific features by representing molecules as graphs, where atoms are nodes and bonds are edges. Graph convolutions applied to these explicit molecular representations have achieved unprecedented accuracy in ADMET property prediction [31]. Feature selection methods can help determine relevant properties for specific classification or regression tasks, alleviating the need for time-consuming experimental assessments [32]. Filter methods are employed during the pre-processing stage to select features from the dataset without relying on any specific machine learning algorithm. These methods swiftly identify and eliminate duplicated, correlated, and redundant features, making them highly efficient in computational terms [33]. They excel at isolating individual features for evaluation, which proves beneficial when features operate independently. However, they fall short in addressing multicollinearity, as they do not mitigate the interdependencies between features. Despite their speed and cost-effectiveness, filter methods may not capture the potential performance enhancements achievable through feature combinations [34]. In a study by Ahmed and Ramakrishnan [35], correlation-based feature selection (CFS), a type of filter method to identify fundamental molecular descriptors for predicting oral bioavailability. Out of 247 physicochemical descriptors from 2279 molecules, 47 were found to be major contributors to oral bioavailability, as confirmed by the logistic algorithm with a predictive accuracy exceeding 71 %.

Wrapper methods, also known as greedy algorithms, iteratively train the algorithm using subsets of features. These methods dynamically add and remove features based on insights gained during previous model training iterations [36]. Unlike filter methods, wrapper methods offer an optimal feature set for model training, leading to superior accuracy. However, their computational demands are higher compared to filter methods due to the iterative nature of the process [33].

In embedded methods, the feature selection algorithm is integrated into the learning algorithm, possessing inherent feature selection capabilities. The models combine filtering and wrapping techniques to optimize feature selection. Initially, these models utilize a filter-based approach to reduce the feature space dimensionality. Subsequently, the best subset of features identified through the filter-based step is incorporated using a wrapper technique [37]. Embedded methods combine the strengths of filter and wrapper techniques while mitigating their respective drawbacks. They inherit the speed of filter methods while surpassing them in accuracy [38].

### 2.4. Molecular descriptors

Molecular descriptors (MD) are crucial components in in-silico research, serving as numerical representations that accurately convey the structural and physicochemical attributes of compounds based on their 1D, 2D, or 3D structures [39]. Various software tools are available for the calculation of molecular descriptors, facilitating the extraction of relevant features for predictive modelling. Table 2 provides a comprehensive list of software packages commonly used in cheminformatics and computational drug discovery for descriptor generation. These programs offer a wide array of over 5000 descriptors, encompassing constitutional descriptors as well as more intricate 2D and 3D descriptors that capture various geometric, connectivity, and physicochemical properties [40]. The choice of molecular representation determines whether a molecule is described using experimental descriptors or theoretical descriptors. Experimental descriptors encompass all measurements obtained through experiments, such as the octanol-water partition coefficient, molar refractivity, polarizability, and various other physicochemical properties obtained through specific experimental procedures [41]. Conversely, theoretical molecular descriptors span 0D, 1D, 2D, 3D and 4D molecular descriptors, which are derived from defined chemoinformatic algorithms applied to a clear molecular representation [42]

**Table 2.** List of software packages for the calculation of Molecular descriptors

Name	Organization/institution	Availability
RDKit	GitHub	https://github.com/rdkit
PaDELPy	University of Massachusetts Lowell	https://github.com/ecrl/padelpy
ADMET Predictor	Simulations Plus, Inc	https://www.simulations-plus.com/
CODESSA™	Semichem	http://www.semichem.com/codessa/default.php
DRAGON	Talete SRL	https://www.talete.mi.it/products/dragon_description.htm
EPISUITE™	United States Environmental Protection Agency	https://www.epa.gov/tsca-screening-tools/epi-suitetm-estimation-program-interface
MOE	Chemical Computing Group	https://www.chemcomp.com/Products.htm
Molconn-Z™	EduSoft	http://www.edusoft-lc.com/molconn/
MOLD2	National Center for Toxicological Research	https://www.fda.gov/science-research/bioinformatics-tools/mold2
MOLGEN	University of Bayreuth	https://www.molgen.de/
PowerMV	National Institute of Statistical Sciences	https://www.niss.org/research/software/powermv
Alvadesc	Alvascience	https://www.alvascience.com/alvadesc/
CORAL	Mario Negri Institute for Pharmacological Research	http://www.insilico.eu/coral/SOFTWARECORAL.html

The 0D molecular descriptors are straightforward and convenient to compute and interpret because they don't require structural information or connectivity between atoms. They are independent of molecular conformation and optimization [43]. While they offer limited information content, they still play a vital role in modelling various physicochemical properties or contributing to more complex models [44]. Descriptors that compute information from fractions of a molecule fall into the 1D molecular descriptors category, often represented as fingerprints - binary vectors where 1 indicates a substructure's presence and 0 its absence [45]. Similar to 0D descriptors, they're straightforward to calculate, interpretable, and conformation-independent [46]. Two-dimensional descriptors describe properties computable from 2D molecular representations, relying on graph theory and maintaining theoretical properties through isomorphism. They're sensitive to molecular characteristics like size, shape, and chemical information [46]. They're divided into structural-topological indices, encoding adjacency and distance, and topochemical indices, quantifying topology and atomic properties [47]. Three-dimensional descriptors relate to the 3D representation of molecules, incorporating molecular conformations, bond distances, angles, and dihedral angles to describe stereochemical properties [39]. Popular types include pharmacophore representations, characterizing steric and electronic features crucial for interactions with biological targets [48]. Grid-based descriptors, also called 4D, introduce a fourth dimension to capture interactions between molecules, their conformations, and biological receptor active sites. By considering ligand conformational variation and interactions within binding pockets, they aim to enhance quantitative structure-activity relationship (QSAR) model reliability [49].

## 3. Machine learning applications in predicting ADME properties

### 3.1. Absorption prediction

Machine learning models have shown promise in predicting intestinal absorption and permeability of compounds. Various approaches have been employed, including artificial neural networks for oligopep-tides [50], support vector machines for general compounds [51], and ensemble methods combining support vector machines (SVM), random forest (RF), and gradient boosting for natural products [52] (A summary of machine learning-based models for absorption prediction is presented in Table 3.). These models have demonstrated high predictive accuracy, with support vector machines achieving up to 91.54 % accuracy [51]. Computational models based on molecular descriptors, such as polar surface area, have also been developed to predict intestinal permeability [53]. These in silico methods offer quick and cost-effective alternatives to experimental techniques like Caco-2 cell assays.

**Table 3.** Summary of machine learning-based models for absorption prediction

No. of comp.	Target	Descriptors	Modelling method	Performance	Ref.
1242	HIA	1D and 2D molecular descriptors	SVM	Accuracy: Training set = 90.38 %; Test set = 91.54 %; MCC = 0.80, AUC = 0.885	[51]
67	HIA	1D - 3D theoretical descriptors plus one of Abraham’s solvation param.	MARS	Whole data set: RMSE = 7.2 % Whole data set: *R*^2^ = 0.93	[54]
31	*K*_a_	MOE descriptors	XGBoost	Training set: RMSE = 0.0023 h^−1^ Prediction set: RMSE = 0.0021 h^−1^	[52]
160	HIA	0D - 3D Dragon theoretical descriptors	Multilayer perceptron-artificial neural network, SVM	Training set: *R*^2^= 0.8; RMSE = 0.18 Test set: *R*^2^= 0.66; RMSE = 0.21	[55]
552	HIA	Adriana Code and Cerius2 0D - 2D theoretical descriptors	Genetic algorithm, partial least squares regression, SVM	Training set: *R*^2^= 0.66; RMSE = 12.5 Test set: *R*^2^= 0.77; RMSE = 16	[56]
1593	HIA	1D - 2D theoretical descriptors	SVM	Accuracy: Training set = 98.5 %, Test set = 99 %	[57]
970	HIA	2D - 3D descriptors, molecular fingerprints and structural fragments	Random forest	Training set: SE = 0.89; SP = 0.85; Q = 0.89 Test set: SE = 0.88; SP = 0.81; Q = 0.87	[58]

HIA - human intestinal absorption; SVM - support vector machines; MCC - Matthews correlation coefficient; AUC - area under the curve; MARS - multivariate adaptive regression splines; RMSE- root mean square error; *K*_a_ - absorption rate constant; SE - sensitivity; SP - specificity; Q - accuracy

The ability to predict intestinal absorption and permeability can significantly aid in drug development by facilitating the selection of promising candidates and potentially reducing attrition rates in clinical trials [52]. Bei *et al.* [59] developed an XGBoost model to predict the subcutaneous absorption rate constant of monoclonal antibodies using only their primary sequence. Kamiya *et al.* [60] used machine learning to estimate key physiologically based pharmacokinetic model parameters, including absorption rate constants, for 212 diverse chemicals. Karalis [61] applied machine learning techniques to identify the maximum plasma concentration (*C*_max_) / time to reach *C*_max_ (*T*_max_) ratio as a potentially superior metric for absorption rate in bioequivalence studies. Kumar *et al.* [62] implemented a graph convolutional neural network model to predict 18 early ADME properties across an enterprise-wide drug discovery pipeline. An integrated strategy combining multiple machine learning models and physiologically-based pharmacokinetic models achieved promising results in predicting human oral bioavailability directly from chemical structure [63]. Artificial Neural Networks outperformed support vector machines in predicting food effects on bioavailability, with key factors including octanol water partition coefficient, hydrogen bond donors, topological polar surface area, and dose [64]. Graph Neural Networks with transfer learning have also shown potential in predicting oral bioavailability, outperforming previous studies by automatically extracting important features from molecular structures [65]. These studies demonstrate the potential of machine learning to enhance the prediction of absorption kinetics and other pharmacokinetic parameters, potentially accelerating drug development processes.

### 3.2. Distribution prediction

This summary examines models for predicting drug distribution in the body, focusing on blood-brain barrier (BBB) penetration and volume of distribution. Physiologically-based pharmacokinetic (PBPK) models are commonly used to predict tissue: plasma partition coefficients and volume of distribution [66]. Key factors affecting BBB penetration include permeability-surface area product (PS), unbound fraction in plasma (*f*_u_, plasma), and brain tissue (*f*_u_, brain) [67] (see Table 4 for an overview of machine learning-based models for distribution prediction). In vivo methods, such as brain microdialysis and in situ brain perfusion, are valuable for assessing brain drug distribution [68].

**Table 4.** Summary of machine learning-based models for distribution prediction

No. of comp.	Target	Descriptors	Modelling method	Performance	Ref.
529	*K*_p_	Fragmental descriptors	Feed-forward back propagation neural network	Q = 0.815, RMSEcv = 0.318	[69]
6741	PPB	2D, 3D, and fingerprints	Consensus of K-nearest neighbours, support vector regression, RF, boosted trees and gradient boosting regressor	MAE = 0.089, RMSE = 0.153; *R*^2^= 0.738	[70]
21	*K*_p_	Not explained	BIOiSIM	Test set: AFE = 0.96 (*C*_max_), 0.89 (AUC), 0.69 (Vd_ss_); AAFE = 1.20 (*C*_max_), 1.30 (AUC), 1.71 (Vd_ss_); *R*^2^= 0.99 (*C*_max_), 0.98 (AUC), 0.99 (Vd_ss_)	[71]
227	PPB	Constitutional descriptors, topological descriptors, geometric descriptors, molecular properties and RDF descriptors	QSAR, convolutional neural network, feed-forward neural network	Training set: MAE= 0.066, *R*^2^= 0.905, MSE= 0.011 Test set: MAE= 0.068, *R*^2^= 0.945, MSE= 0.007	[72]
1970	*K*_p_	Daylight fingerprints, atomic and ring multiplicities, simple molecular parameters and chemical descriptors	RF, SVM	Overall accuracy of 95 %, mean square contingency coefficient (*>*) of 0.74	[73]
310	BBB	Topological descriptors, geometrical descriptors, electrostatic and quantum chemical descriptors	SVM, genetic algorithm- partial least squares	Training set: *R*^2^ = 0.98, RMSE = 0.117 Test set: *R*^2^ = 0.98, RMSE = 0.118	[74]
208	*K*_p_	Constitutional descriptors, topological descriptors, geometric descriptors, electrostatic descriptors and quantum chemical descriptors	Least squares SVM	Training set: *R*^2^ = 0.97, RMSE = 0.0226 Test set: *R*^2^ = 0.97, RMSE = 0.0289	[75]

*K*_p_ - tissue-to-plasma partition coefficient; PPB - plasma protein binding; RF -random forest, MAE -mean absolute error; MSE - mean square error; AUC - area under the curve; AFE -average fold error; AAFE - absolute average fold error; Vd_ss -_ volume of distribution at steady-state.

A simple two-descriptor model using molecular volume and polar surface area has been proposed for predicting BBB penetration [67]. Overall, current methods predict drug volume of distribution with an average 2-fold error [66]. Rapid brain equilibration requires high BBB permeability and low brain tissue binding [67], highlighting the importance of considering multiple drug design and selection factors. Iwata *et al.* [76] developed ML models for total body clearance and steady-state volume of distribution (Vd using imputed nonclinical data, achieving accuracies comparable to animal scale-up models. Parrott *et al.* [77] integrated ML-predicted properties into physiologically-based pharmacokinetic (PBPK) models for highly lipophilic compounds, showing promising results for *V*_d_ predictions. Antontsev *et al.* [71] demonstrated a hybrid approach combining ML optimization with mechanistic modelling to predict tissue-plasma partition coefficients accurately. Mulpuru and Mishra [78] developed an AutoML model for predicting plasma unbound fraction, achieving a coefficient of determination of 0.85. Cao *et al.* [79] created a model to classify Polyfluorinated alkyl substances (PFAS) binding fractions in plasma, with 92 % accuracy. Riedl *et al.* [80] introduced a descriptor-free deep learning model using bidirectional encoder representations from transformers (BERT) for predicting plasma unbound fraction, offering flexibility and minimal domain expertise requirements. These studies demonstrate the potential of machine learning in predicting drug binding and distribution properties.

### 3.3. Metabolism prediction

Machine learning techniques have shown promising results in predicting drug metabolism and interactions related to cytochrome P450 (CYP) enzymes. Various approaches, including k-nearest neighbours, decision trees, random forests, artificial neural networks, and support vector machines, have been employed to classify CYP activities and predict interactions with high accuracy [81] (For a structured overview of ML models in drug metabolism, see Table 5). Machine learning has also been applied to model relationships between chemical structure and metabolic fate, addressing complex endpoints such as metabolic stability and in vivo clearance [82].

**Table 5.** Summary of machine learning-based models for metabolism prediction

No. of comp.	Target	Descriptors	Modelling method	Performance	Ref.
4545	Metabolic pathway	Molecular fingerprints, physicochemical properties, structural descriptors	Graph convolutional network and RF	Single-class classification 95.16 % Multi-class classification 97.61 %	[83]
1917	Metabolic pathway	Physicochemical properties and others	RF	On external test: ACC 0.74, MCC 0.48, Sensitivity 0.70, Specificity 0.86, PPV 0.94, NPV 0.46	[84]
26138	Metabolic Stability	2D descriptors	Principal component analysis, XGBoost	Test set: ACC 93.6 %	[85]
16613	Cytochrome inhibition	2D descriptors	RMSE, XGBoos	Test set: ACC 97.6 %	[86]
(Substrate, inhibitor) data: CYP1A2-(396, 13459) CYP2C9-(518, 12677) CYP2C19-(628, 13162) CYP2D6-(714, 13732) CYP3A4-(1584, 12990)	Metabolic DDIs	2D descriptors, CATS, ECFP4 and MACCS	RF, XGBoos	Internal validation: ACC 0.8, AUC 0.9 External validation: ACC 0.795 Multi-level validation: ACC 0.793	

PPV - positive predictive value; NPV - negative predictive value; ACC - accuracy

Recent advancements have led to the development of consensus models for predicting metabolic drug-drug interactions (DDIs) related to five important CYP450 isozymes (CYP1A2, 2C9, 2C19, 2D6, 3A4), achieving high accuracy and robustness in both internal and external validations [86]. These in silico methods have become valuable tools in drug discovery and development, offering efficient alternatives to time-consuming and costly experimental assessments. Mamada *et al.* [87] developed a novel combination model using DeepSnap-Deep Learning and conventional ML, achieving high accuracy in predicting rat clearance. Keefer *et al.* [88] compared ML and mechanistic in vitro-in vivo extrapolation (IVIVE) models, finding that ML IVIVE models performed comparably or better than mechanistic counterparts for human intrinsic clearance prediction. Rodríguez-Pérez *et al.* [89] introduced a multitask graph neural network architecture for multispecies intrinsic clearance prediction, approaching experimental variability in performance. Andrews-Morger *et al.* [90] explored ML strategies to improve rat clearance predictions for physiologically based pharmacokinetic modelling, demonstrating enhanced accuracy compared to standard in vitro bottom-up approaches. These studies highlight the potential of ML models in improving clearance predictions across species, contributing to more efficient drug discovery and development processes.

### 3.4. Excretion prediction

Machine learning models have emerged as powerful tools for predicting drug metabolism and excretion in early drug discovery and development [91]. Researchers have developed in-silico prediction systems for renal excretion and clearance, incorporating factors such as the fraction unbound in plasma to improve accuracy [92]. ML models for pharmacokinetic (PK) prediction complement established approaches like IVIVE and PBPK models [93]. Ongoing research focuses on improving model accuracy, addressing limitations, and integrating ML approaches into drug discovery workflows to enhance efficiency and clinical success rates [93]. Recent studies have explored machine learning approaches for predicting pharmacokinetic parameters, including clearance (CL), elimination rate constant (*k*_e_), and half-life (t_½_) (Table 6 outlines key machine learning models developed for predicting drug excretion parameters). Seal *et al.* [94] created PKSmart, an open-source model for predicting human PK parameters, including volume of distribution at steady-state (VDss), CL, and *t*_½_, using molecular fingerprints and animal PK data. Fan *et al.* [95] focused on predicting drug half-life using ensemble and consensus machine learning methods, with XGBoost outperforming other individual models and a consensus model further enhancing prediction performance. These studies demonstrate the potential of machine learning approaches in improving the accuracy and efficiency of PK parameter prediction in drug discovery and development.

**Table 6.** Summary of machine learning-based models for excretion prediction

No. of comp.	Target	Descriptors	Modelling method	Performance	Ref.
244	Intrinsic clearance	Molecular fingerprints, physico-chemical properties, and 3D quantum chemical descriptors	Partial least squares, RF, multi-label classification, principal component analysis	*R*^2^ = 0.96, *Q* = 48	[96]
748	Total clearance	The chemical structure was represented as graph	Deep learning	Test data set: Geometric mean fold error = 2.68	[97]
1114	Total clearance	2D SMARTS-based descriptors Model building using - StarDrop	RF, radial basis function	Whole data set: *R*^2^ = 0.55, RMSE=0.332	[98]
112	Intrinsic clearance	233 molecular descriptors	Artificial neural network	Training set: *R*^2^ = 0.953, RMSE = 0.236 Test set: *R*^2^ = 0.804, RMSE = 0.544	[99]
349	Renal clearance	195 descriptors	Partial least squares, RF	Training data: *R*^2^ =0.93, RMSE = 0.32 Test data: *R*^2^ = 0.63, RMSE = 0.63	[100]
1352	Renal clearance	2D and 3D descriptors and 49 fingerprints	SVM, gradient boosting machine, XGBoost, RF	Training set: *R*^2^ = 0.882, RMSE = 0.239 Test set: *R*^2^ = 0.875, RMSE = 0.103	[101]

## 4. Machine learning approaches in toxicity prediction

### 4.1. In silico toxicity models

Machine learning models have become increasingly popular for predicting various toxicity endpoints, including hepatotoxicity, nephrotoxicity, cardiotoxicity, and genotoxicity [102]. Various ML models for predicting drug toxicity are summarized in Table 7. These models utilize physicochemical properties and in vitro assays to predict drug-induced liver injuries (DILI), which are major causes of drug attrition [103]. Support vector machines and random forests are among the most commonly used algorithms for toxicity prediction [104]. Khan *et al*. [104] developed an ensemble model integrating ML and deep learning algorithms, achieving 80.26% accuracy in predicting hepatotoxicity. Ancuceanu *et al.* [105] used the DILIrank dataset to create 78 models, which were then stacked for improved performance. Lu *et al.* [106] focused on predicting hepatotoxicity of drug metabolites using an ensemble approach based on support vector machines, achieving 78.47 % balanced accuracy. For specific drugs like colistin, machine learning models using electronic health records have been developed to predict nephrotoxicity, identifying key risk factors and dose thresholds [107].

**Table 7.** Summary of machine learning-based models for toxicity prediction

No. of comp.	Target	Descriptors	Modelling method	Performance	Ref.
Mice = 6,226 Rat = 6,238	Acute oral toxicity	Molecular fingerprints, molecular descriptors	Graph neural network, RF, SVM, artificial neural network	Accuracy: Mice = 0.9586; Rat = 0.9335 MCC: Mice = 0.5514; Rat = 0.4929 AUROC: Mice = 0.7778; Rat = 0.7442	[108]
575	Hepatotoxicity	1D and 2D molecular descriptors	RF	Accuracy= 0.631	[109]
7889	Cardiotoxicity	MOE and Mol2vec descriptors	Multitask A	AUC: training set = 0.944; validation set= 0967	[110]
641	Genotoxicity	Molecular fingerprints, molecular descriptors	SVM, RF	Accuracy = 0.937	[111]
6512	Mutagenicity	Molecular fingerprints, molecular descriptors	SVM	AUC= 0.93	[112]
863	Carcinogenicity	Mol2vec, Mold2, MACCS	Deep learning	MCC= 0.432	[113]

AUROC - area under the receiver operating characteristic curve

A radiomics-based ML model for predicting radiotherapy-induced cardiotoxicity in breast cancer patients demonstrated high performance, with AUC up to 97 % when combining dosimetric, demographic, clinical, and imaging features [114]. Another study introduced cardioToxCSM, a web-based tool that predicts six types of cardiac toxicity outcomes for small molecules, achieving AUC values up to 0.898 [115]. The MultiFlow^®^ DNA damage assay (MFA), which measures four mechanistic markers at two time points, has been combined with ML to enhance genotoxicity assessment and predict the mode of action of DNA-damaging agents [116]. Recent studies have achieved high accuracies in predicting genotoxicity, with models reaching up to 95 % accuracy on training data and 92 % on external test sets [116]. These studies demonstrate the potential of ML in toxicity prediction. The availability of large toxicology databases has facilitated the development of more accurate models [117]. However, challenges remain, such as the need for benchmarking datasets due to inconsistencies in toxicity assignments across different sources [102]. Despite these challenges, computational toxicology has made significant progress over the past decade, with machine learning models showing promise in predicting various toxicity endpoints and potentially reducing the need for costly and time-consuming in vivo studies [102].

### 4.2. Adverse drug reactions

Machine learning approaches have shown promising potential in predicting toxicological properties and adverse drug reactions (ADRs) of pharmaceutical agents [118]. Recent advancements include the development of AI models for precise prediction of compound off-target interactions, which can be used to differentiate drugs and classify compound toxicity [102]. The MAESTER framework integrates diverse features to predict tissue-specific adverse events with high accuracy, sensitivity, and specificity [119]. Similarly, the Off-targetP ML framework uses deep learning and automated machine learning to predict off-target panel activities directly from compound structures, aiding in drug design and discovery [120]. These computational methods offer efficient, low-cost tools for early assessment of compound safety and toxicity, potentially reducing costly failures in drug development and identifying toxic [102].

### 4.3. Machine learning for predicting dose-dependent toxicity

Machine learning is revolutionizing toxicology by enhancing predictive capabilities across various dosing ranges and improving safety assessments. ML models can analyse large datasets to predict drug toxicity, environmental hazards, and off-target effects, offering more efficient and accurate risk evaluations [121-123-87]. These models can be applied early in drug discovery to identify potential safety liabilities and filter out problematic compounds [117]. The integration of ML with DNA-encoded libraries (DELs) shows promise for modelling binding to off-targets and improving predictive toxicology [123]. Various toxic endpoints, including acute oral toxicity, hepatotoxicity, and cardiotoxicity, can be predicted using ML methods, although performance varies depending on the dataset and chemical space covered [117]. Despite challenges, ML in toxicology offers significant potential for enhancing risk assessment, determining clinical toxicities, and detecting harmful side effects of medications [121].

## 5. Overview of common machine learning techniques in ADME-Tox prediction

ML is a method of data analysis involving the development of new algorithms and models capable of interpreting a multitude of data [14]. The algorithms used in recent years have successively improved their performance with the increase in both the quantitative and qualitative aspects of data available for learning [124]. Figure 2 illustrates commonly used AI/ML algorithms for developing ADMET prediction models. ML is considered one of the best options available when applied to solve problems for which a big amount of data and various variables are available to the individual, but a model or formula relating these various variables amongst themselves, along with the expected result, is not known [3,4]. However, when drug discovery moved into an era of a large amount of data, ML approaches evolved into DL approaches, which are more powerful and efficient in dealing with the massive amounts of data generated from modern drug discovery approaches [125]. DL is a subset of ML based on artificial neural networks that use multiple layers to progressively extract higher-level features from raw input. Due to its ability to learn from data and the environment, DL and neural network (NN), also known as artificial neural networks (ANN) named after its artificial representation of the working of a human nervous system, have become one of the most successful techniques in various AI research areas [126].

Different types of machine learning models with varying degrees of complexity can predict molecular properties, such as similarity-based models, linear models, kernel-based models, Bayesian models, tree-based models, and neural networks.

### 5.1. Traditional machine learning approaches

#### 5.1.1. Random forest

Random forest (RF) is widely used for ADMET due to its ability to handle high-dimensional data and complex, non-linear relationships. Random forest is an ensemble learning method that utilizes multiple decision trees to make predictions. It operates by constructing a multitude of decision trees during training and outputting the mode of the classes (classification) or the mean prediction (regression) of the individual trees [127]. RF introduces randomness both in the selection of data points used to build each tree and in the selection of features used at each split point. This randomness helps to decorrelate the trees, making the ensemble more robust and less prone to overfitting [128]. It has been used to predict toxicity endpoints [109], metabolic stability [84], and solubility [129]. RF has emerged as a powerful machine learning technique for predicting absorption, distribution, metabolism, excretion, and toxicity (ADMET) properties of compounds. RF models have been successfully applied to classify toxicity datasets [130] and predict maximum recommended daily pharmaceutical doses [131]. These models utilize substructure fingerprints as descriptors, which encode the presence or absence of specific molecular substructures. RF's ability to identify important substructure features provides insights into structure-toxicity relationships [130]. The predictive performance of RF models can be further improved through rigorous model selection processes, as demonstrated in the Tox21 Challenge [132]. RF has also been employed in computational studies to evaluate ADMET properties of natural products, such as Papua red fruit flavonoids, aiding in the assessment of their potential as bioactive compounds in functional foods [133]. These applications highlight RF's versatility and effectiveness in ADMET prediction and toxicity evaluation.

#### 5.1.2. Support vector machines

Support vector machines (SVMs) are effective for binary classification tasks in ADMET modelling, especially for toxicology studies (like classifying compounds as toxic or non-toxic). SVMs have emerged as a powerful tool in predicting ADMET properties in drug discovery. It works by finding the optimal hyperplane that best separates different classes or predicts the continuous target variable by maximizing the margin between the classes [121]. SVMs can handle both linear and nonlinear data by using appropriate kernel functions. SVM can be extended to handle nonlinear data by mapping the input features into a higher-dimensional space using a kernel function [134]. The kernel function computes the dot product between the feature vectors in the higher-dimensional space without explicitly transforming the data. Common kernel functions include linear, polynomial, radial basis function (RBF), and sigmoid kernels, which allow SVM to capture complex nonlinear relationships in the data. SVMs, along with other machine learning techniques like Random Forests and Decision Trees, have become dominant methods in predictive toxicology due to their ability to handle complex datasets [121]. Studies have shown that SVMs can accurately classify compounds based on human intestinal absorption, using molecular descriptors such as topological polar surface area and predicted octanol-water distribution coefficient. SVMs have demonstrated competitive performance compared to other state-of-the-art techniques in pharmaceutical classification tasks [135]. However, challenges remain in fully integrating predictive ADMET modelling into drug discovery processes, and there is a need for larger, high-quality datasets and improved molecular descriptors to fully realize the potential of machine learning techniques in this field [136].

#### 5.1.3. K-nearest neighbours

The k-nearest neighbours (k-NN) algorithm has shown promise in predicting various aspects of absorption, distribution, metabolism, excretion, and toxicity (ADMET) properties of chemicals. K-nearest neighbour is a simple and intuitive supervised learning method used for classification and regression tasks. It operates on the principle that objects (*e.g.* data points) with similar characteristics are often found near each other in the feature space. The k-NN algorithm classifies or predicts the label of a new data point by considering the labels of its k-nearest neighbours, where k is a user-defined parameter [137]. Studies have demonstrated its effectiveness in predicting sub-chronic oral toxicity in rats [138], acute contact toxicity of pesticides in honeybees [139], and chronic toxicity based on acute toxicity data [140]. These k-NN models have achieved reasonnable accuracy, with external validation results ranging from 65 to 77 % for different endpoints. The approach has been valuable in prioritizing chemical safety assessments and potentially reducing animal testing [139]. Incorporating ADMET screening earlier in the drug discovery process has become crucial for identifying poorly behaved compounds and improving the success rate of new chemical entities reaching the market. These computational models can play a significant role in predicting toxicity and supporting future risk assessments.

#### 5.1.4. Gradient boosting machines and extreme gradient boosting

Recent studies have demonstrated the effectiveness of gradient boosting algorithms in predicting Absorption, Distribution, Metabolism, Excretion, and Toxicity (ADMET) properties of drug compounds. Tian *et al.* [141] developed ADMET boost, a web server utilizing extreme gradient boosting (XGBoost) for accurate ADMET prediction, achieving top rankings in multiple benchmark tasks. An *et al.* [142] compared various machine learning models, including XGBoost and light gradient boosting machines (LGBM), for predicting estrogen receptor alpha (ERα) bioactivity and ADMET properties, finding high accuracy and robustness in their approach. Li *et al.* [143] applied LGBM to predict ADMET properties of anti-breast cancer compounds, reporting superior performance compared to other algorithms. These studies highlight the potential of gradient boosting techniques in drug discovery and development, offering accurate predictions of crucial pharmacokinetic and toxicological properties. The integration of such models into web-based tools further enhances their accessibility and utility in the biopharmaceutical field.

### 5.2. Deep learning approaches

#### 5.2.1. Fully Connected neural networks

Fully connected neural networks (FCNNs) and other artificial neural network architectures have shown significant potential in predicting ADMET properties of chemical compounds. These models can achieve high accuracy in predicting various toxicological parameters, with some studies reporting accuracies between 74 and 98% [144]. FCNNs have demonstrated superior performance in multitask learning approaches, improving predictive quality for properties like human metabolic stability [145]. Neural networks outperform traditional methods in predicting several ADMET properties, including acute toxicity, carcinogenicity, and hepatic clearance [144]. The application of neural networks in ADMET prediction allows for early consideration of these properties in drug development, potentially reducing late-stage failures and improving pharmaceutical industry efficiency [146]. As artificial intelligence continues to advance, neural networks are expected to play an increasingly important role in toxicology research and drug discovery [147].

#### 5.2.2. Convolutional neural networks

Convolutional neural networks (CNNs) and other deep learning models have shown promising results in predicting absorption, distribution, metabolism, excretion, and toxicity (ADMET) properties of drug candidates. These models can analyse large datasets to identify relevant features and evaluate hidden trends among multiple ADMET parameters [145]. Deep neural networks have demonstrated superior performance compared to traditional methods in predicting properties such as microsomal stability, passive permeability, and log *D* [148]. Various ADMET prediction models have been developed and made publicly available, offering rapid assessments of important drug properties like cytotoxicity, mutagenicity, and drug-drug interactions [149]. The integration of ADMET screening earlier in the drug discovery process helps eliminate poorly behaved compounds, reducing costly failures in later stages[5]. As artificial intelligence continues to advance, neural networks are expected to play an increasingly important role in toxicology research and the development of accurate biosensors for toxic substance detection [147].

#### 5.2.3. Recurrent neural networks and long short-term memory

Recent research has explored the application of recurrent neural networks (RNNs) and long short-term memory (LSTM) models in predicting and analysing ADMET properties of drugs and peptides. Wang *et al.* [15] demonstrated that LSTM networks can accurately model complex pharmacokinetic-pharmacodynamics relationships . Wenzel *et al.* [145] developed multitask deep neural networks for predicting multiple ADME-Tox properties simultaneously, showing improved performance over single-task models. González-Díaz [150] reviewed multi-output QSPR models for predicting ADMET processes, including drug-target interactions and nanoparticle toxicity. In the realm of peptide design, Müller *et al.* [151] utilized LSTM RNNs to generate novel antimicrobial peptide sequences with a higher predicted activity rate compared to randomly sampled sequences. These studies highlight the potential of RNN and LSTM models in drug discovery, ADMET prediction, and peptide design, offering promising tools for pharmaceutical research and development.

#### 5.2.4. Graph neural networks

Recent advancements in Graph neural networks (GNNs) have significantly improved the prediction of ADMET properties in drug discovery. De Carlo *et al.* [152] developed an attention-based GNN that processes molecular information from substructures to whole molecules, effectively predicting ADMET properties without relying on molecular descriptors. Aburidi and Marcia [153] introduced an optimal transport-based graph kernel approach that outperformed state-of-the-art GNNs on multiple ADMET datasets. Feinberg *et al.* [31] demonstrated that graph convolutions applied to explicit molecular representations achieve unprecedented accuracy in ADMET prediction, enabling both interpolation and extrapolation to new chemical spaces. Wenzel *et al.* [145] explored multitask deep neural networks for ADME-Tox modelling, showing improved performance compared to single-task models and introducing a "response map" visualization technique for interpreting model predictions. These studies collectively highlight the potential of GNNs and deep learning approaches to enhance ADMET property prediction in drug development.

### 5.3. Generative models for ADMET-optimized molecule design

#### 5.3.1. Variational autoencoders and generative adversarial networks

Variational autoencoders (VAEs) and generative adversarial networks (GANs) are powerful machine learning models with applications in various fields. Adversarial Variational Bayes (AVB) unifies VAEs and GANs, allowing for more expressive inference models [154]. The connection between VAEs, GANs, and minimum Kantorovitch estimators has been explored from an optimal transport perspective [155]. In the field of drug development and toxicology, multi-output QSPR models have been used to predict absorption, distribution, metabolism, excretion and toxicity (ADMET) properties for drugs, pollutants, and nanoparticles [150]. ADME profiling has significantly reduced pharmacokinetic drug failures in clinical trials and has become crucial for safety and toxicity prediction across industries [156]. The integration of ADME information with in vitro results and computer modelling is essential for developing quantitative in vitro to in vivo extrapolations and integrated testing strategies in toxicology [156].

#### 5.3.2. Reinforcement learning (RL)

Generative AI models combined with reinforcement learning can efficiently design drug candidates with suitable ADMET properties [157]. Quantum-informed molecular representation learning has improved ADMET property prediction, achieving state-of-the-art results in multiple tasks [158]. The REMEDI framework uses reinforcement learning to model bile acid metabolism adaptations in primary sclerosing cholangitis, demonstrating potential for exploring treatments [159]. RL is emerging as a powerful tool in drug discovery and development, offering potential to accelerate and optimize the process. RL algorithms can improve sample efficiency and policy optimization for de novo drug design [160]. These approaches enable simultaneous optimization of molecules for multiple goals through structure-based drug design and high-throughput screening; one such example is where RF has been applied to optimize failed anticancer drugs by considering multiple properties simultaneously, including binding affinity and toxicity profiles [161].

## 6. Recent developments in ADMET modeling

### 6.1. Integration of machine learning methods with physiologically-based pharmacokinetic and quantitative structure-activity relationship models

Physiologically-based pharmacokinetic (PBPK) models are mathematical representations of how chemicals enter the body through various routes such as inhalation, ingestion, or dermal exposure. These models describe how much of the chemical enters the bloodstream, its distribution among different tissues, and how the body metabolizes and eliminates it [162]. They incorporate information about the body's anatomy, physiology, and biochemical processes. PBPK models can range from simple versions with few features to complex ones that capture intricate details about chemical movement and fate in the body [163]. However, creating PBPK models for new chemicals is challenging due to their complexity and the numerous parameters involved [163]. To address this, some researchers have proposed an integrated approach that combines a simplified PBPK model with machine learning based quantitative structure-activity relationship (QSAR) models [164]. This integrated approach aims to estimate plasma and tissue concentrations, as well as various pharmacokinetic (PK) parameters, by leveraging databases containing in vivo and in vitro data along with the structural and physicochemical properties of selected compounds. ML models are trained using these databases to predict ADME parameters, which are then incorporated into the PBPK model to simulate time-concentration profiles and calculate PK parameters such as Area under the Curve (AUC) and maximum concentration (*C*_max_). The performance of the integrated ML-based PBPK model is assessed against in vivo PK data, and if satisfactory, the model can be used to generate simulation data for further refinement and validation [165].

This approach aims to enhance efficiency in drug development and reduce animal testing [166]. A novel computational platform combining ML and PBPK models has demonstrated improved accuracy in predicting pharmacokinetic profiles without experimental data, potentially accelerating early drug discovery [167]. Additionally, adapting PBPK models for ML applications has shown promise in recapitulating summary pharmacokinetic parameters, although limitations in the underlying PBPK models may affect prediction accuracy [169]. Despite challenges such as the need for diverse training data and improved interpretability of ML models, the integration of ML/AI approaches with PBPK modelling is expected to facilitate more efficient and robust ADMET predictions for a wide range of chemicals [165].

### 6.2. Generalization

#### 6.2.1. Multi-task learning

While each ADMET parameter can be studied independently, they are interconnected and influence each other throughout the drug development process. Therefore, ADMET is not a sequential process but rather a holistic approach that considers the interplay between absorption, distribution, metabolism, excretion, and toxicity to predict drug behaviour accurately [14]. Considering this, it would be practical to develop a reliable multitask model that could simultaneously predict multiple ADME endpoints. Despite the advancements made by classical single-task learning in predicting individual ADMET endpoints using abundant labelled data, multi-task learning (MTL) emerges as a promising paradigm, offering a solution that reduces reliance on endpoint labels while simultaneously predicting multiple ADMET endpoints [169]. By leveraging shared information and underlying correlations between different ADMET properties, MTL provides a more comprehensive and efficient framework for predictive modelling in ADMET studies. For example, Wenzel *et al.* [145] introduced an industrialized approach for optimizing deep neural network (DNN) models to predict ADME-Tox properties, utilizing up to 50,000 compounds from diverse databases. The study highlights the significance of DNN hyperparameters and molecular descriptors in model success, demonstrating the superiority of multitask DNNs in predictive performance across various datasets. For instance, multitask DNNs showed improved predictive quality compared to single-task models, with an increase in R2 from 0.6 to 0.7 for human metabolic stability data in external validation sets. In another study, S. Zhang *et al.* [170] employ machine learning techniques, including random forest (RF) and artificial neural network (ANN), to develop multi-task (MT) models for predicting tissue-to-blood partition coefficient (Ptb) values across various mammalian tissues. Compared to single-task (ST) models, the MT approach consistently outperforms, with ANN-based MT models exhibiting the highest prediction accuracy, showcasing determination coefficients ranging from 0.704 to 0.886 and low root mean square errors and mean absolute errors across multiple endpoints. The study by Walter *et al.* [171] investigates multi-task machine learning models for predicting ADME and animal PK endpoints using in-house data of 28 endpoints. It reveals the superior performance of multi-task graph-based neural networks, attributing this success to the influence of endpoints with larger data sets, such as physicochemical endpoints and microsomal clearance.

#### 6.2.2. Transfer learning

Developing highly accurate models is essential for swiftly evaluating pharmacokinetic (PK) properties during the drug development process. Traditional models, which rely on a substantial amount of existing domain knowledge and constructing these models is rather time-consuming; thus, with transfer learning, which aims to generalize the knowledge gained from one task to another task to enhance its applicability and decision-making. Leveraging the common features learned from a similar source domain, transfer learning has been demonstrated to be able to develop the models without learning from scratch [172]. Ye *et al.* [173] introduced an integrated transfer learning and multitask learning approach utilizing three deep neural networks to predict pharmacokinetic parameters. The consensus model, DeepPharm utilized transfer learning from three pre-trained deep neural networks: DeepPharm-BA, DeepPharm-PPBR, and DeepPharm-VDss&HL and achieved the highest accuracies of 27.78, 44.22, 63.33 and 68.39 % in predicting oral bioavailability (BA), plasma protein binding rate (PPBR), apparent VDss and elimination half-life (HL), respectively, outperforming conventional machine learning methods. In a study proposed by Abbasi *et al.* [174] explores the transfer of knowledge across different physiological and biophysical domains, evaluating its effectiveness in predicting compound activity with limited labelled data. By leveraging source datasets such as Tox21, ToxCast, SIDER, HIV, and BACE, the proposed approach transfers knowledge between related or semi-related tasks, demonstrating improved performance in target tasks. Additionally, the study highlighted the importance of selecting appropriate source datasets and revealed that knowledge transfer between tasks within the same category yields better results compared to tasks from different categories. In another study S. Wang et al. [175] introduced a semi-supervised model named SMILES-BERT for molecular property prediction, utilizing deep learning techniques and a large-scale of unlabelled data. The model utilized an attention mechanism-based Transformer Layer and underwent pre-training via a Masked SMILES Recovery task on the ZINC dataset. This pre-training process significantly improved the model's generalization capability, as evidenced by achieving an exact recovery rate of 82.85 % on the validation dataset. During fine-tuning, the model was trained using various learning rates and optimization strategies, resulting in high prediction performance across three datasets: log *P*, PM2, and PCBA-686978. SMILES-BERT surpassed state-of-the-art methods, highlighting its ability to effectively leverage unlabelled data and its potential for molecular property prediction tasks with varying dataset sizes and properties. Similarly, X. Li and Fourches [176] introduced MolPMoFiT, an inductive transfer learning method for molecular activity prediction in Quantitative Structure-Activity Relationship (QSAR) modelling. The approach utilized a pre-trained Molecular Structure Prediction Model (MSPM) using one million unlabelled molecules from ChEMBL, fine-tuning it for specific QSAR tasks. This method achieved strong performance across four benchmark datasets (lipophilicity, FreeSolv, HIV, and blood-brain barrier penetration), when compared to state-of-the-art techniques reported in the literature. The approach showcased its potential for improving next-generation QSAR models, particularly for smaller datasets with challenging endpoints.

#### 6.2.3. Pretrained models

Recent advancements in machine learning have significantly improved the prediction of absorption, distribution, metabolism, excretion, and toxicity (ADMET) properties in drug discovery. Pretrained models and self-supervised learning approaches have shown promising results in this field. Zhang *et al.* [177] developed HelixADMET, a system incorporating self-supervised learning that achieved a 4 % improvement over existing ADMET systems. Jung *et al.* [178] utilized the pretrained ChemBERTa model for ADMET prediction, exploring various architectures. Wenzel *et al.* [179] demonstrated the effectiveness of multitask deep neural networks in predicting ADME-Tox properties, showing improved performance compared to single-task models. Kumar *et al.* [62] implemented an enterprise-wide predictive model, gTPP, which outperformed commercial ADME models and automatic model builders. These studies highlight the potential of advanced machine learning techniques, particularly pretrained models and self-supervised learning, in enhancing ADMET property prediction and facilitating early-stage drug development.

### 6.3. Interpretable and explainable ADMET models

As artificial intelligence and machine learning models grow in complexity, a significant challenge has surfaced within the field: the absence of transparency and interpretability [180]. While these models demonstrate remarkable predictive capabilities, elucidating the rationale behind their predictions remains difficult. This absence of interpretability can pose challenges for researchers and regulatory authorities in relying on AI and ML-driven predictions, particularly in drug discovery [157]. Moreover, assessing and prioritizing discovered targets or compounds becomes cumbersome without understanding the decision-making process of AI algorithms. In order to foster trust in AI/ML systems, it is imperative that models are transparent and understandable to users thus efforts are being made to enhance the interpretability by embracing explainable artificial intelligence (XAI), which tries to offer clear and intelligible justifications for the predictions made by AI and ML models of machine learning models in ADME studies [181]. Interpretability in AI exposes the inner workings of these systems, allowing for the detection of issues like information leakage, model bias, robustness, and causality [182].

#### 6.3.1. Local interpretable model-agnostic explanations and Shapley additive explanations

Recent research has explored the application of explainable artificial intelligence (XAI) techniques, particularly LIME (Local Interpretable Model-agnostic Explanations) and SHAP (SHapley Additive exPlanations), in interpreting complex machine learning models for medical applications such as Alzheimer's disease detection [183]. The paper introduced LIME, a method designed to provide interpretable explanations for complex machine learning models by approximating their behaviour locally. LIME achieves this by generating local surrogate models around specific instances, enabling users to understand model predictions on individual data points. A novel extension, KG-LIME, has been developed to predict individualized risk of adverse drug events in multiple sclerosis therapy, leveraging knowledge graphs for more interpretable explanations [184]. Another study by Gabbay *et al.* [185] presents a LIME-based explainable machine learning model for predicting the severity level of COVID-19 diagnosed patients. By employing LIME, the model provides interpretable insights into the factors influencing severity prediction, aiding in understanding and decision-making in COVID-19 management.

Another explainable technique called SHAP (SHapley Additive exPlanations) methodology has emerged as a powerful tool for interpreting machine learning models in ADMET prediction and drug design. SHAP enables the identification and prioritization of molecular features that influence compound activity and potency predictions, regardless of model complexity [186]. This approach has been applied to various ADMET properties, including metabolic stability [187] and general ADME profiles [188]. SHAP analysis can be used to interpret predictions from diverse machine learning algorithms, such as random forests, support vector machines, and deep neural networks [189]. By providing insights into the contribution of specific structural features to model outcomes, SHAP aids in compound optimization and supports experts in drug candidate selection [188]. This interpretability enhances confidence in machine learning applications within pharmaceutical research.

These advancements demonstrate the growing importance of interpretable machine learning models in drug discovery and optimization, offering researchers valuable tools for understanding and improving ADMET predictions.

## 7. Machine learning techniques in clinical trial designs

In silico prediction of absorption, distribution, metabolism, and excretion (ADME) properties, as well as model-informed drug discovery and development (MID3)1 strategies. MID3 includes providing quantitative predictions for aspects such as pharmacokinetics, pharmacodynamics, efficacy and safety end points, and disease progression [190]. By leveraging these models, researchers can optimize dosing strategies, inform clinical trial designs, and obtain robust quantitative assessments regarding drug efficacy and safety. The mainstay of modelling activities for drug development includes an empirical compartmental model built from sparsely sampled PK/PD datasets [191]. In this respect, AI/ML provides new ways for pharmacometricians to think about their models.

There have been a number of approaches proposed in using feed-forward NNs [192] for modelling of PK(/PD) data. However, these did not tackle the more complex problem of extrapolating outside the range of observed data. In fact, the main limitation of such models is that they do not explicitly encode causality relationships among dose, PKs, and PDs and, hence, cannot enable robust predictions of new dosing regimens.

In the 1990s, the availability of biological reagents and liquid chromatography mass spectrometry dramatically reduced the attrition of small-molecule drugs due to PK considerations. Currently, attrition due to poor clinical exposure is rare, with preclinical toxicology, clinical intolerability, or insufficient efficacy being the major sources of attrition [7]. Reagents, such as microsomes, cryopreserved hepatocytes, recombinant drug metabolizing enzymes, and cells overexpressing specific transporters, have enabled drug metabolism and PK departments to generate large quantities of in vitro ADME data over the last 15 to 20 years. These data serve two specific functions: first, in vitro data related to metabolic stability, plasma protein binding, permeability, efflux, and CYP inhibition can be used for the design (*i.e.* prior to synthesis) of small molecules with superior ADME properties, along with other parameters, such as biochemical and cellular potency and selectivity data; second, archived data can be used to build ML models to predict these properties (In silico optimization).

## 8. Data sources and challenges in machine learning based ADME-Tox prediction

### 8.1. Key databases for ADME-Tox data

The development of predictive models for ADME-Tox properties is crucial in drug discovery, but it relies heavily on the availability and quality of data. Several databases and resources have emerged to address this need. Canault *et al.* [193] proposed an interactive network of databases to facilitate finding relevant ADME-Tox data sources. Ekins and Williams [194] advocated for making preclinical ADME-Tox data freely available on the web, suggesting the expansion of databases like ChemSpider. Pawar *et al.* [195] conducted a comprehensive review of over 900 databases relevant to in silico toxicology, categorizing them based on various criteria. To assist in compound filtering, Miteva *et al.* [196] developed FAF-Drugs, an online service that allows users to process their compound collections using simple ADME-Tox filtering rules. These resources collectively aim to improve drug development processes by enhancing access to and utilization of ADME-Tox data.

### 8.2. Data quality and availability issues

Data quality issues have become increasingly critical in the era of big data, affecting various domains and applications [197]. These issues encompass multiple dimensions, including accuracy, completeness, consistency, and currency [198]. Poor data quality can significantly impact organizational efficiency and decision-making, leading to financial losses and credibility issues [198]. Data cleaning, a crucial process in addressing these problems, is particularly important when integrating heterogeneous data sources and in data warehouse environments [200]. Researchers have proposed various methodologies and techniques to tackle data quality challenges, drawing from fields such as data mining, probability theory, and machine learning [201]. These approaches often involve the use of data quality rules and algorithms for detecting and correcting errors, as well as managing issues like data deduplication and information completeness [198].

### 8.3. Integration of multi-omics data

Multi-omics data integration is crucial for understanding complex biological systems and improving clinical outcomes. Various strategies have been developed, including early, mixed, intermediate, late, and hierarchical integration approaches [202]. Machine learning algorithms have been applied to multi-omics data to produce diagnostic and classification biomarkers [203]. Tools like OmicsNet use multilayer networks to integrate heterogeneous omics data, facilitating functional analysis, biomarker discovery, and drug response prediction [204]. The integration of multi-omics data has applications in disease subtyping, biomarker prediction, and deriving biological insights [205]. Despite progress in the field, challenges remain in developing computational methods for the proper integration of multi-omics datasets [204]. Researchers have developed numerous software tools and methods to address these challenges and improve clinical outcome predictions [205].

### 8.4. Privacy and ethical concerns

Privacy and ethical concerns in research and technology adoption have gained significant attention. Key issues include ensuring meaningful user notice, access control, data anonymization, and algorithm validation to prevent harm [206]. The increasing use of technology in learning processes raises concerns about learner tracking, necessitating principles for trust, accountability, and transparency in learning analytics [207]. RFID technology, while promising for data collection, presents challenges for privacy due to its ability to track individual products [208]. Researchers are exploring situations where privacy may not always be optimal, considering the balance between privacy and other competing values in research and design [209]. These studies emphasize the importance of addressing ethical and privacy concerns in various contexts, from social-behavioural research to technological implementations, to ensure responsible data use and protect individuals' rights.

## 9. Case studies of machine learning in ADME-Tox prediction

### 9.1. Successful machine learning applications in drug development

Machine learning techniques are increasingly applied across various stages of drug discovery and development to accelerate the process and reduce failure rates [3]. ML approaches have shown promise in target validation, biomarker identification, and digital pathology analysis [3]. Specific applications include SNP discoveries, drug repurposing, virtual screening, lead identification, QSAR modelling, and ADMET analysis [210]. Algorithms such as support vector machines, random forests, and artificial neural networks have demonstrated success in predicting human intestinal absorption and identifying novel compounds for cancer treatment [210]. The Janssen gTPP model, employing graph convolutional neural networks, has shown superior performance in predicting early ADME properties compared to commercial models [62]. However, challenges remain in the interpretability and repeatability of ML-generated results, necessitating systematic data generation and validation of ML approaches [3,4].

### 9.2. Real-world use cases

Machine learning techniques have been increasingly applied to predict ADME-Tox properties of drug candidates, helping to streamline the drug discovery process and reduce costs [211]. These computational methods rely heavily on high-quality experimental data to generate accurate models. Flexible approaches combining multiple technologies, such as Bio-Rad's KnowItAll ADME/Tox system with support vector machine platforms, have shown promise in improving prediction performance and overcoming limitations of individual methods [212]. Despite the potential of in silico approaches, regulatory requirements for ADME and toxicokinetic data vary widely across different chemical frameworks, with some areas having minimal or no requirements [213]. Incorporating ADME/TK information early in toxicity testing can enhance study design, support 4R goals, and ultimately improve risk assessment and characterization of chemical safety [213].

### 9.3. Drug repurposing applications

Drug repurposing, the process of using existing drugs for new indications, has gained attention due to its potential to reduce costs and development time [214]. This approach leverages existing ADMET data to expedite drug development [215]. Chemical structure modifications and nanotechnology applications can improve ADME-Tox properties of drug candidates, enhancing absorption, permeability, distribution, and stability while reducing toxicity [216]. ABC transporters play a crucial role in ADMET, influencing drug resistance and passage through cellular barriers [217]. Various models and assay systems, including in vitro, in vivo, and in silico approaches, can be used to analyze drug interactions with ABC transporters and predict ADMET profiles [217]. These strategies collectively contribute to more efficient drug discovery and development processes, potentially leading to improved therapeutic outcomes.

## 10. Challenges and limitations in machine learning based ADME-Tox prediction

### 10.1. Data scarcity and imbalance

Machine learning techniques have been increasingly applied to ADME-Tox prediction, offering promising tools for toxicity screening and compound profiling [211]. However, data scarcity and class imbalance pose significant challenges in developing accurate models. Studies have shown that class imbalance can significantly impact model performance, particularly affecting recall and F1 scores [218]. To address these issues, various strategies have been explored, including resampling methods and transfer learning. Resampling techniques have demonstrated improvements in sensitivity and specificity for nuclear receptor profiling [219]. Additionally, transfer learning approaches have shown success in predicting drug activity and toxicity for targets with insufficient data by leveraging information from data-rich targets [220]. These methods, along with appropriate evaluation metrics and hyperparameter tuning, can enhance the performance of toxicity classification models and improve predictions for understudied targets [221].

### 10.2. Model interpretability and trustworthiness

Data scarcity and imbalance pose significant challenges for deep learning models, particularly in high-stakes domains [222]. These issues can lead to reduced model performance and trustworthiness. To address data scarcity, various techniques have been proposed, including transfer learning, self-supervised learning, and generative adversarial networks [222]. For imbalanced datasets, interpretable machine learning approaches can help identify class prototypes, sub-concepts, and outlier instances [223]. However, class imbalance can adversely affect the stability of interpretation methods like LIME and SHAP, particularly in credit scoring applications [224]. When evaluating model interpretability and trustworthiness, it's crucial to consider the inductive bias of different algorithms. For instance, in generalized additive models (GAMs), tree-based approaches offer a good balance of sparsity, fidelity, and accuracy, making them potentially more trustworthy [225].

### 10.3. Generalization across chemical space

Machine learning models in chemistry often face challenges due to data scarcity and imbalance, which can lead to overfitting and poor generalization. Several strategies have been proposed to address these issues. Farthest point sampling in chemical feature spaces can generate well-distributed training datasets, enhancing model performance across various algorithms [226]. Latent space enrichment, combining disparate data sources in joint prediction tasks, improves prediction in data-scarce applications [227]. Similarity-based machine learning enables on-the-fly data selection and model training for specific queries, requiring only a fraction of data to achieve competitive performance [228]. When dealing with class imbalance and data scarcity in toxicity classification models, appropriate resampling algorithms, evaluation metrics, and hyperparameter tuning are crucial for optimal performance [218]. These approaches collectively offer promising solutions to enhance machine learning model performance in chemistry, particularly when faced with limited or imbalanced datasets.

### 10.4. Model validation and regulatory acceptance

Data scarcity and class imbalance pose significant challenges in developing and validating machine learning models, particularly for safety-critical applications. These issues can affect model performance metrics and potentially invalidate underlying assumptions [229]. Studies have shown that class imbalance significantly impacts recall and F1 scores, while hyperparameter tuning can improve performance on imbalanced datasets [218]. To address data scarcity, various techniques have been proposed, including transfer learning, self-supervised learning, and generative adversarial networks [222]. For regulatory acceptance of models, it is crucial to consider model domain, uncertainty, validity, and predictability [230]. Researchers emphasize the importance of using appropriate evaluation metrics, tuning hyperparameters, and ensuring the trustworthiness of training datasets [222]. These considerations are essential for developing reliable and effective models in fields such as toxicity prediction, aviation, and medical imaging.

## 11. Future directions and innovations

### 11.1. Integrating AI with experimental approaches

Recent research highlights the integration of AI with experimental approaches in various fields. AI and ML models can serve as fast surrogates for time-consuming experiments or computational models, enhancing predictive capabilities while reducing data requirements [231]. In molecular design, AI techniques are being combined with experimental validation and chemistry automation, although these efforts are still in early stages [232]. The development of cell therapies is benefiting from AI and ML methods, which can generate predictive models and design rules based on high-throughput screening data [233]. The integration of AI with geography, termed GeoAI, is providing novel approaches for addressing environmental and societal problems [234]. Future directions include the automatic discovery of physical laws, active learning for optimal experiment design, and the integration of multi-fidelity data from various computational models and experimental instruments [231].

### 11.2. Advances in explainable AI

Explainable AI (XAI) is an emerging field aimed at increasing the interpretability and transparency of machine learning models. Recent research highlights the integration of diverse approaches to advance XAI. Experimental psychology methods can contribute to XAI by applying cognitive modelling techniques to artificial black boxes [235]. Formal methods, such as algebraic decision diagrams, can enhance explainability by providing precise characterizations of AI outcomes [236]. The arts offer valuable contributions to address limitations in explainable AI, fostering collaborations between scientists and artists to investigate human-machine entanglements [237]. Current XAI approaches in deep learning encompass various applications, evaluation metrics, and challenges, with ongoing research focusing on improving trust, accountability, and interoperability of complex neural networks [238]. These multidisciplinary efforts aim to create more transparent and understandable AI systems across diverse domains.

### 11.3. Potential of quantum computing and machine learning

Recent research explores the synergies between quantum computing, AI and ML. Quantum computing shows significant potential in revolutionizing drug discovery and development. It offers faster and more accurate molecular characterization through quantum simulation, outperforming classical quantum chemistry methods [239]. Quantum machine learning (QML) algorithms are emerging as strong competitors to classical approaches, particularly in the early stages of drug discovery for identifying novel drug-like molecules [240]. QC's ability to perform complex calculations efficiently could accelerate the drug discovery process, making it more cost-effective and accurate [241]. Recent applications of QC in drug development include protein structure prediction, molecular docking, quantum simulation, and quantitative structure-activity relationship models [242]. While current quantum devices are still susceptible to noise and errors, hybrid quantum-classical approaches and quantum-inspired devices like quantum annealers have demonstrated quantum advantage [242]. Further research and development are needed to fully leverage QC's potential in drug discovery [241].

### 11.4. Regulatory and industry trends

Artificial intelligence is revolutionizing drug discovery and preclinical research by integrating with experimental approaches. AI techniques, such as machine learning and neural networks, are improving the efficiency and effectiveness of drug candidate identification and optimization [243]. The integration of virtual and experimental screening methods, including high-throughput screening and DNA-encoded libraries, is enhancing early-phase drug discovery [243]. AI combined with new experimental technologies is expected to make drug discovery faster, cheaper, and more effective [244]. Additionally, integrated approaches to testing and assessment (IATA) are being developed to replace animal testing in toxicology, incorporating in vitro, in-silico, and in vivo methods [245]. While AI-driven approaches offer significant benefits, challenges remain, such as the need for high-quality databases and addressing regulatory requirements [245].

## 12. Conclusion

The application of artificial intelligence and machine learning in absorption, distribution, metabolism, excretion and toxicity (ADMET) predictions has revolutionized drug discovery and development. Traditional in vitro and in vivo approaches, though essential, are often time-consuming, costly, and sometimes unreliable in predicting human responses. AI and ML-driven computational methods provide a faster, more accurate, and cost-effective alternative by leveraging large-scale datasets and predictive modelling techniques such as deep learning, support vector machines, and ensemble learning.

Molecular descriptors, ranging from 0D to 4D, alongside feature selection methods like filter, wrapper, and embedded techniques, play a crucial role in refining model accuracy and interpretability. These advancements facilitate high-throughput virtual screening, enabling the early identification of drug candidates with favourable pharmacokinetic and toxicity profiles, thereby reducing the likelihood of late-stage failures. Additionally, deep learning architectures have shown significant promise in predicting complex metabolic and toxicity pathways.

However, challenges persist, including data quality, model generalizability, and regulatory acceptance. Standardized datasets, improved interpretability of AI models, and rigorous validation protocols are necessary for broader adoption. Regulatory agencies must establish clear guidelines for integrating AI-driven ADMET predictions into drug development workflows.

Moving forward, integrating AI-driven predictions with experimental validation will be key to optimizing drug development pipelines. By overcoming current limitations and fostering collaboration between computational and experimental research, AI and ML can drive more efficient, precise, and cost-effective drug discovery, ultimately leading to safer and more effective therapeutics.

## ABBREVIATIONS

ADMET: Absorption, distribution, metabolism, excretion and toxicity
ADRs: Adverse drug reactions
ANN: Artificial neural network
AVB: Adversarial variational bayes
BA: Bioavailability
BBB: Blood brain barrier
BERT: Bidirectional encoder representations from transformers
CFS: Correlation-based feature selection
CL: Clearance
CNNs: Convolutional neural networks
CYP: Cytochrome
DDIs: Drug-drug interactions
DELs: DNA-encoded libraries
DILI: Drug-induced liver injuries
DNN: Deep neural network
ERα: Estrogen receptor alpha
FCNNs: Fully connected neural networks
Fu: Unbound fraction
GANs: Generative Adversarial networks
GNNs: Graph neural networks
IATA: Integrated approaches to testing and assessment
IVIVE: *in vitro-in vivo* extrapolation
*K*_e_: Elimination rate constant
k-NN: k-nearest neighbours
LSTM: long short-term memory
MD: Molecular descriptors
ML: Machine Learning
MTL: Multi-task learning
NN: Neural network
PBPK: Physiologically-based pharmacokinetic
PFAS: Polyfluorinated alkyl substances
PK: Pharmacokinetics
PS: Permeability-surface
QML: Quantum machine learning
QSAR: Quantitative structure-activity relationship
RBF: Radial basis function
RF: Random forest
RNNs: Recurrent neural networks
SVM: Support vector machines
VAEs: Variational autoencoders
*V*_d_: Volume of distribution
XAI: Explainable artificial intelligence
